# Why are they not coming back? A single-center follow-up study on oncological women oocyte’s storing for fertility preservation

**DOI:** 10.3389/fendo.2022.1054123

**Published:** 2022-12-06

**Authors:** Valentina Immediata, Federico Cirillo, Annamaria Baggiani, Maria Federica Zanagnolo, Camilla Ronchetti, Emanuela Morenghi, Amalia Cesana, Cristina Specchia, Paolo Emanuele Levi-Setti

**Affiliations:** ^1^ Department of Gynecology, Division of Gynecology and Reproductive Medicine, Fertility Center, Humanitas Research Hospital, IRCCS, Milan, Italy; ^2^ Biostatistics Unit, Humanitas Research Hospital, IRCCS, Milan, Italy; ^3^ Department of Biomedical Sciences, Humanitas University, Milan, Italy

**Keywords:** fertility preservation, cancer, cryopreserved oocytes, ICSI outcome, follow-up

## Abstract

**Introduction:**

Oocyte cryopreservation is a valid option for female cancer patients to preserve fertility. The number of patients undergoing fertility preservation (FP) cycles has increased over the past years. Nevertheless, the rates of patients returning to use their cryopreserved material have shown to be considerably low, ranging from 5-8%, but significant data regarding the reasons of such low return rates are scarce.

**Methods:**

This study is a single-center follow-up retrospective study evaluating the return rate of oncological women who underwent FP at a tertiary care Fertility Center and assessing the reasons influencing the patients who did not return. Data about patients who returned to attempt pregnancy were retrieved from internal registries. Non-returned patients were assessed with a standardized phone survey investigating health condition, marital status and family projects, spontaneous conceptions, and the reasons why they had not returned to use their gametes. A univariate analysis between returned and non-returned patients was performed.

**Results:**

Of the 397 patients who received counseling about FP, 171 (43.1%) underwent oocyte cryopreservation between 2001 and 2017. Nine (5%) died, and 17 (10%) were lost at follow-up. A total of 20 patients (11.7%) returned and 125 did not. In the non-returned group, 37 (29.6%) did not have a partner, 10 (8%) had a previous spontaneous conception, and 15 (12%) had recurrent malignancy at the time of follow-up. In the univariate analysis, younger age at freezing (31.8±6.2 vs. 35.2±4.7; p 0.018), lack of a partner (p 0.002), type of cancer (other than breast cancer; p 0.024) were the significant factors in the non-returned group. As for the personal reason for not coming back, patients mainly answered as follows: lack of a partner (29, 23.2%), the desire for spontaneous motherhood (24, 19.2%), previous spontaneous pregnancies after FP procedures (16, 12.8%), and still ongoing hormonal therapy for breast cancer (13, 10.4%). All patients confirmed their will to keep the storage of their oocytes.

**Discussion:**

The impact of a cancer diagnosis on a woman’s maternal desire, sentimental status and life priorities should be studied more thoroughly. Studies investigating hormonal therapy suppression in breast cancer patients seeking pregnancy should be encouraged.

**Clinical trial registration:**

https://clinicaltrials.gov, identifier NCT05223764.

## Introduction

The recent technological evolutions of Assisted Reproductive Technologies (ART), together with the growing recognition of the impact of a potential fertility loss on patients undergoing cancer therapy have motivated the expansion of the fertility preservation (FP) field (1, 2).

Patient education regarding future reproductive function is an important component of the care of individuals with cancer and it has been reported that receiving counseling about reproductive loss and the option to try to preserve fertility before treatment is important to survivors, even if they are unable to have children after chemotherapy (3, 4).

Since the development of vitrification, cryopreservation of mature oocytes has proven its efficacy in egg banking programs for both medical indications and age-related fertility loss (5, 6). Though the literature is rich in studies on oocyte cryopreservation, reports on its efficacy are much more sporadic than those reporting on the use of fresh oocytes. This is especially the case for patients undergoing FP for medical indications. Most of these studies are retrospective (5, 7), and only a few available report prospective data on fertility preservation for medical indications (8). Altogether, evidence sustains oocyte cryopreservation as an effective and safe practice for these patients, even though long-term data on children born after treatment are yet unavailable. Although extensive literature on the importance of FP has been established (7–10), detailed information on patients’ future decisions and on return rates is scarce. Especially when oocyte cryopreservation is concerned, the available data has often been collected from stand-alone clinics and fertility groups (11–14) or cross-sectional surveys and interviews (7, 15–17). This limitation particularly concerns oncological patients, making it hard to have a comprehensive perspective on the matter. Still, it is possible to retrieve valuable information from the few available studies. In general, in both non-medical and oncological settings, the demand up-take of oocyte cryopreservation appears to be increasing each year (7, 9). Nonetheless, the number of women returning to thaw and use their oocytes has not revealed such a significant increase (18).

A study published in 2019 by Humanitas Fertility Center provided data on the outcomes achieved by 244 women who had undergone oocyte cryopreservation for oncological reasons over an eighteen-year period (9). At that time of the survey, only 4.5% had returned to use their stored material, after an average interval period of 3.4 years (9). Similar results had been previously observed in male cancer patients in the same center (19). Also in 2019, Rodriguez-Wallberg and colleagues published a prospective cohort study on a long-term follow-up after fertility preservation where 8% of cancer patients returned to attempt pregnancy with their stored oocytes (8). Motivations of non-return were not investigated by the authors. Cobo and colleagues have documented similar results in cancer patients’ return rate compared to non-medical FP patients (7.4% vs. 12.1%), and a longer storage time (mean storage time 4.1 ± 0.9 vs. 2.1 ± 1.6 years) (7). As possible explanations for the reduced return rate they suggested the long time needed for cancer patients to overcome their disease and, given their relatively younger age (mean age = 32.3 ± 3.5 years vs. 7.2 ± 4.9 years), their possible higher probability of getting pregnant by natural conception (7). In addition, the long-lasting regimens of endocrine treatments have been hypothesized to be an added cause of delay in breast cancer patients (7). It is important for clinicians to improve the come-back rate working on the modifiable factors, such as the psychological aspects of delaying the childbearing. Indeed, even though the tumor has been recovered, many patients still actively avoid pregnancy for fear of recurrence, of the eventual complications of pregnancy after having suffered from a severe disease, of eventual cancer inheritance in the offspring. Misconceptions on these topics may influence patients’ choice and negatively affect their serenity during child seeking or pregnancy. Moreover, physicians often showed concerns about starting FP medications before chemotherapy and did not feel adequately trained on the safety and timing of these therapies. Oncologists and gynecologists should discuss pregnancy issues with patients when starting medications in order to provide appropriate information; therefore, pre-conception counselling on an individualized basis should be mandatory for all patients of reproductive age to reassure them that obtaining disease remission and facing with the eventual obstetrical risks is possible. A psychological support may play a fundamental role in these patients, such as an improved awareness in clinicians, both oncologists and gynecologists.

This study aims at evaluating the return rate of oncological women who underwent fertility preservation through oocyte cryopreservation at Humanitas Research Hospital’s Fertility Center and assessing the reasons influencing patients who did not return.

## Materials and methods

### Study design and population

This is a single-center follow-up study, performed between January 2020 and July 2021, of all women who underwent oocyte cryopreservation cycles for oncological purposes from January 2001 to December 2017 at the Fertility Center of Humanitas Research Hospital, Rozzano (Milano), Italy, a university-affiliated tertiary care ART center.

The Humanitas Fertility Center employs a standard operating procedure for fertility preservation as previous described (9, 20). Referrals for FP were received from internal or external oncologists, who also provided information on the disease stage and the date of initiation of oncological treatment. Patients obtained the first dedicated FP counselling within a few days. Immediate access was granted thanks to specific personnel resources allocations to enable prompt scheduling of treatment for FP, in order not to delay the starting of the planned cancer therapy. The ovarian reserve was evaluated by the antral follicles count, by measuring follicle stimulating hormone (FSH) and, more recently, Anti-Mullerian Hormone (AMH) levels. Generally, patients underwent a gonadotropin releasing hormone (GnRH) antagonist cycle possibly in the early follicular phase of the cycle or randomly. An aromatase inhibitor 5 mg was also prescribed daily (Femara, Novartis, NJ, USA) to patients with hormone-dependent breast cancer during the stimulation period starting from the second day of the induction to 7 days after oocyte retrieval. Final oocyte maturation was triggered by subcutaneous injection of 0.25 mg recombinant hCG (Ovitrelle, EMD Serono, MA, USA) or 0.2 mg Triptorelin (Decapeptyl, Ipsen, France) to decrease the risk of ovarian hyperstimulation syndrome (OHSS), when at least two follicles reached 16 mm in diameter. Ultrasound-guided oocyte retrieval was performed 48 h later under deep sedation.

After retrieval, all oocytes were selected and cryopreserved using the techniques of slow-freezing until 2009 (21) and open vitrification after 2010 as described by Kuwayama et al. (22). Kitazato^®^ (Kitazato, Shizuoka, Japan) provided the vitrification and warming solutions. Differently from the standard procedural protocol where only mature (metaphase II - MII) oocytes were stored, with oncological patients also immature oocytes (germinal vesicle - GV, and metaphase I - MI) have been generally stored, though it is still an experimental method (23).

Annually, patients renew their agreement to store their cryopreserved oocytes by postal mail after receiving a reminder from the Hospital. The renewal is free of charge.

All costs of the procedures were covered by the Italian National Healthcare System, including the Gonadotropins.

### Data collection and follow-up

Data about the oocyte cryopreservation cycles, ART procedures and the information about the oncological history of the patients were retrieved from the Fertility Center’s internal web-based registry (Art-it). Every three months, the data set is regularly updated, including thawed cycles and demises.

Between January 2020 and May 2021, all patients were contacted by phone call and were asked a set of standardized questions (**Table 1**).

**Table 1:** Study’s standardized verbal interview.

Present health conditions
**Current general health**
**Oncological history**
**Type and stage of malignancy** **Cancer treatment (surgery, chemotherapy, radiotherapy, hormonal therapy, immune therapy)** **Relapse**
**Gynecological and obstetric history**
**Pregnancies and births prior to cancer diagnosis** **Conditions/pathologies affecting patient’s fertility** **Menstrual cyclicity and menopausal symptoms after cancer treatment** **Diagnosis of Premature ovarian insufficiency (POI) after cancer treatment** **Sexual disorders after cancer treatment** **Estroprogestinic therapy after cancer treatment** **Spontaneous pregnancies and births after cancer treatment**
**Family projects and sentimental status**
**Sentimental status prior to cancer diagnosis** **Current sentimental status** **Desire of motherhood** **Desire to use cryopreserved oocytes**
**Socio-economic status**
**School degree** **Job and occupational status**

The survey’s purpose was to investigate their present health conditions, cancer treatment and any potential relapses, their family projects and sentimental status, any spontaneous conception, and conditions that may affecting patient’s fertility (such as endometriosis, polycystic ovarian syndrome, chemotherapy/radiotherapy, or gynecological surgery prior to oocyte retrieval). Furthermore, it included the personal reasons why they had not yet returned following FP.

### Ethical approval and data protection

All data were collected anonymously in the exclusive internal web-based database. Patients’ data are safeguarded by advanced threat prevention, enterprise-class encryption, and authentication for any user with the periodical need of password renewal.

Patients included in this study had consented in writing to the usage of their anonymized medical records for research and follow-up purposes, as long as their anonymity was protected, and their medical record’s confidentiality was assured.

The Independent Ethical Committee of Humanitas Research Hospital approved the study protocol (n. 72/20), and the protocol was registered on ClinicalTrials.gov (NCT05223764).

### Statistical analysis

All analyses were carried out using STATA 15.0 (StataCorp. 2017. Stata Statistical Software: Release 15. College Station, TX: StataCorp LP). Patient data were assessed by using multivariate comparisons as well as univariate tests. Data were described as number and percentage, or mean and standard deviation, as appropriated. A p-value lower than 0.05 was considered significant.

## Results

**Figure 1:**
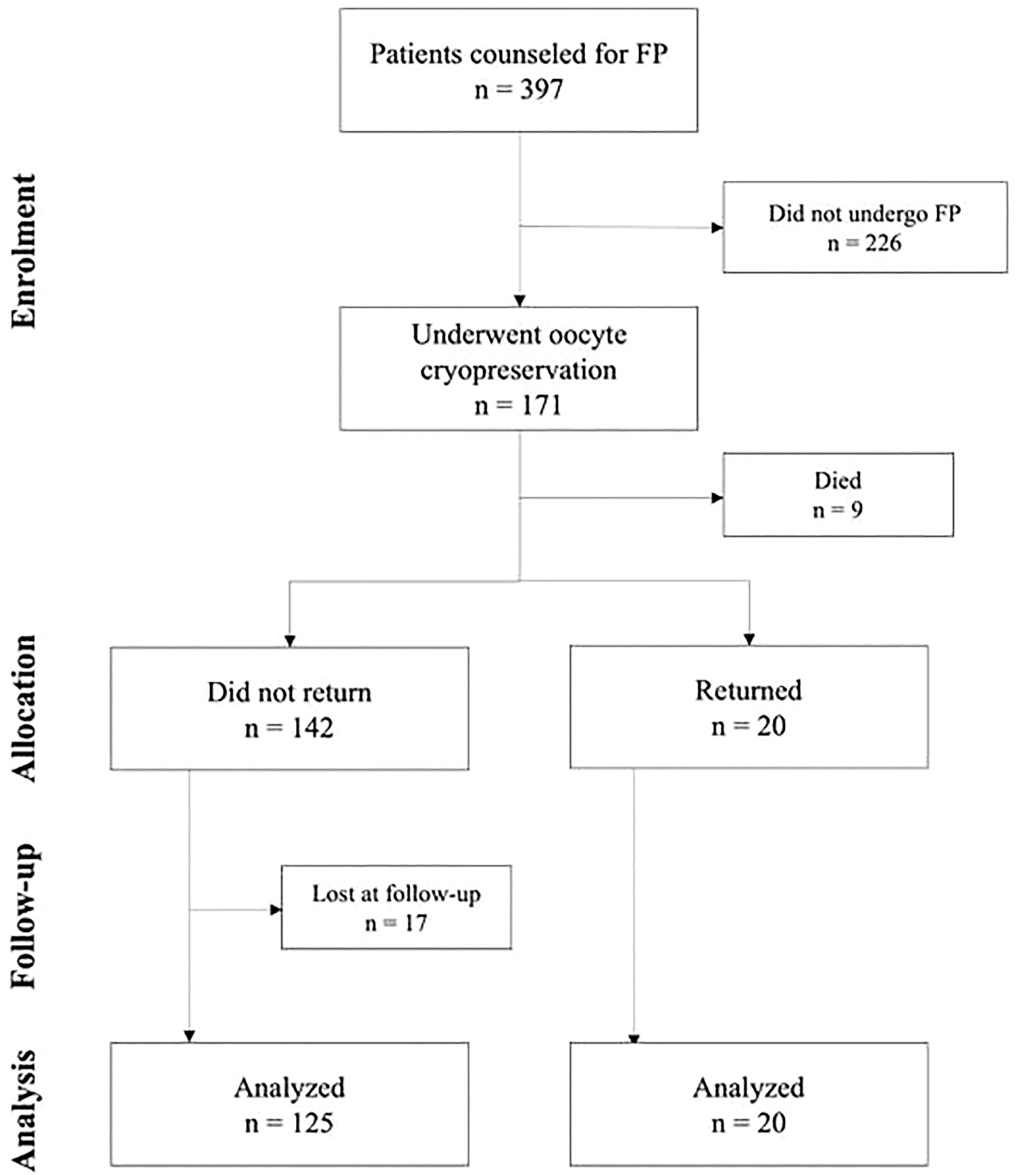
Study’s population flowchart.

Of the 397 oncological patients who received counseling about FP at the Fertility Center of Humanitas Research Hospital, between January 2001 and December 2017, 171 (43.1%) underwent oocyte cryopreservation. At the time of follow-up 20 patients (11.7%) had returned to use their frozen material and 9 (5.3%) had died. Among the 142 non-returned patients, 125 (88.0%) participated in the study’s survey and 17 (12.0%) were lost. The flowchart illustrating the study’s population is shown in **Figure 1**. Of the nine dead patients, 4 (44.4%) had been diagnosed with breast cancer, and 2 (22.2%) with lymphoma. The remaining three suffered from sarcoma, colon cancer, and brain cancer, respectively.

The clinical and sociocultural characteristics of the 145-patient cohort analyzed in this study’s investigation (returned and non-returned groups) are listed in **Table 2**.

**Table 2:** Clinical and sociocultural characteristics of study population.

Variable	All (n = 145)	Returned (n = 20)	Non-returned (n = 125)	p
Age				
At freezing	32.2±6.1 33 (17-45)	35.8 ± 4.1 36 (25-44)	31.8±6.2 32 (17-45)	0.018
At follow-up	38.5 ± 6.6 39.5 (22-64)	39.9 ± 3.9 40.5 (29-46)	37.8±6.9 38 (22-63)	0.050
Cancer type				0.024
Breast	99 (68.3%)	14 (70.%)	85 (68.00%)	
Hematological	37 (25.5%)	2 (10.0%)	35 (28.0%)	
Gynecological	2 (1.4%)	1 (5.0%)	1 (0.8%)	
Other cancers	7 (4.8%)	3 (15.0%)	4 (3.2%)	
Treatment				
Chemotherapy	117 (80.7%)	14 (70.0%)	103 (82.4%)	0.223
Hormonal therapy	81 (55.9%)	13 (65.0%)	68 (54.4%)	0.470
Radiotherapy	99 (68.7%)	9 (45.0%)	90 (72.0%)	0.019
Relapse	15 (10.3%)	0	15 (12.0%)	0.226
School degree				
8^th^ grade	5 (3.4%)	0	5 (4.0%)	
High school	49 (33.8%)	5 (25.0%)	44 (35.2%)	
University	91 (62.8%)	15 (75.0%)	76 (60.8%)	
Employment				
At freezing	138 (95.2%)	20 (100%)	118 (94.4%)	0.345
At follow-up	125 (86.2%)	19 (95.0%)	106 (84.8%)	0.310
With a partner				
At freezing	103 (71.0%)	9 (45.0%)	94 (75.2%)	0.004
At follow-up	108 (74.5%)	20 (100%)	88 (70.4%)	0.002
Already with children at freezing	11 (7.6%)	1 (5.0%)	10 (8.0%)	1.000

By July 2021, 20 patients had returned to use their oocytes after an average interval period of 4.7 (± 2.5) years. None of these patients had recurrent disease.

Patients’ mean age was 35.8 ± 4.1 (range 25-44) years at freezing and 40.0 ± 4.0 (range 29-46) years at thawing. Most patients (n = 14, 70%) had a diagnosis of breast cancer. Since in Italy Law 40/2004 does not allow sperm donation for single women, all patients had a partner when they decided to come back. Those who were already in a relationship at the time of freezing (n = 9, 45.0%) returned with the same partner. The cumulative pregnancy rate (number of pregnancies per couple) was 40%, and the cumulative live-birth rate (number of live-births per couple) was 30%.

Of the 142 patients who did not return to use their stored material, 125 participated in this study’s follow-up. The mean interval period (± SD) between oocyte retrieval and follow-up was of 6.1 (± 2.3) years (range 3-18 years). By comparing this group of patients to the ones who returned to seek a pregnancy, it was possible to observe a younger age at freezing for the former (31.8 ± 6.2 vs. 35.80 ± 4.10 years; p 0.018). Cancer types (other than breast cancer) were a significant factor (p 0.024): a higher proportion of non-returned patients suffered from hematological diseases (28.0% vs. 10.0%), while the proportion of gynecological cancers (5.0% vs. 0.8%) and of other cancer types (i.e., sarcomas, gastric, colon, and brain cancers) (15.0% vs. 3.2%) was higher among patients who attempted a pregnancy. Chemotherapy was administered to 70.0% of returned patients and 82.4% of non-returned ones (p 0.223). Conversely, treatment with radiotherapy was found to be significant, as a higher proportion of non-returned patients had to undertake this therapy (72.0% vs. 45.0%; p 0.019). Hormonal therapy for breast cancer did not show a significant difference between the two groups (65.0% in returned patients vs. 54.4% in non-returned ones; p 0.470). Fifteen patients in the non-returned group had recurrent diseases when the survey was taken, while none of the patients who came back to attempt pregnancy had relapses (12% vs. 0%; p 0.226). For what concerns sociocultural differences between the two groups, the most significant difference among them was the presence of a partner at follow-up: only 70.4% of the non-returned ones were in a relationship (p 0.002). A similar difference, though not as much significant, regarded the presence of a partner at cancer diagnosis: 75.2% of patients who did not return and 45.0% of those who did return had a partner (p 0.004). Employment differences were not significant, both at oocyte freezing and at follow-up. Finally, obstetric history prior to oocyte freezing was not a significant factor: 5.0% of returned patients and 8.0% of non-returned ones (p 1.000) already had children. Thirty-one women (24.8%) did not have a partner when they had their oocytes retrieved, and the number of single patients increased in the years following (37 patients, 29.6%). Of those in a relationship at the time of freezing (n = 94), 18 had become single by the time of follow-up. Ten patients (8.0%) had already had children prior to oocyte freezing, while 15 (12.0%) had a spontaneous conception after surviving their disease. At the time of writing this report, one patient (0.8%) was pregnant from a spontaneous conception. Of the patients who underwent chemotherapy (103, 82.4%), only 64 (62.1%) recovered their physiological menstrual cycles. Altogether, ten patients (8.0%) were diagnosed with premature ovarian insufficiency (POI) in the years following their recovery. Fifteen patients (12.0%) had recurrent malignancy at the time of follow-up: eleven had breast cancer, two had hematological malignancies, and two suffered from sarcoma.

**Figure 2:**
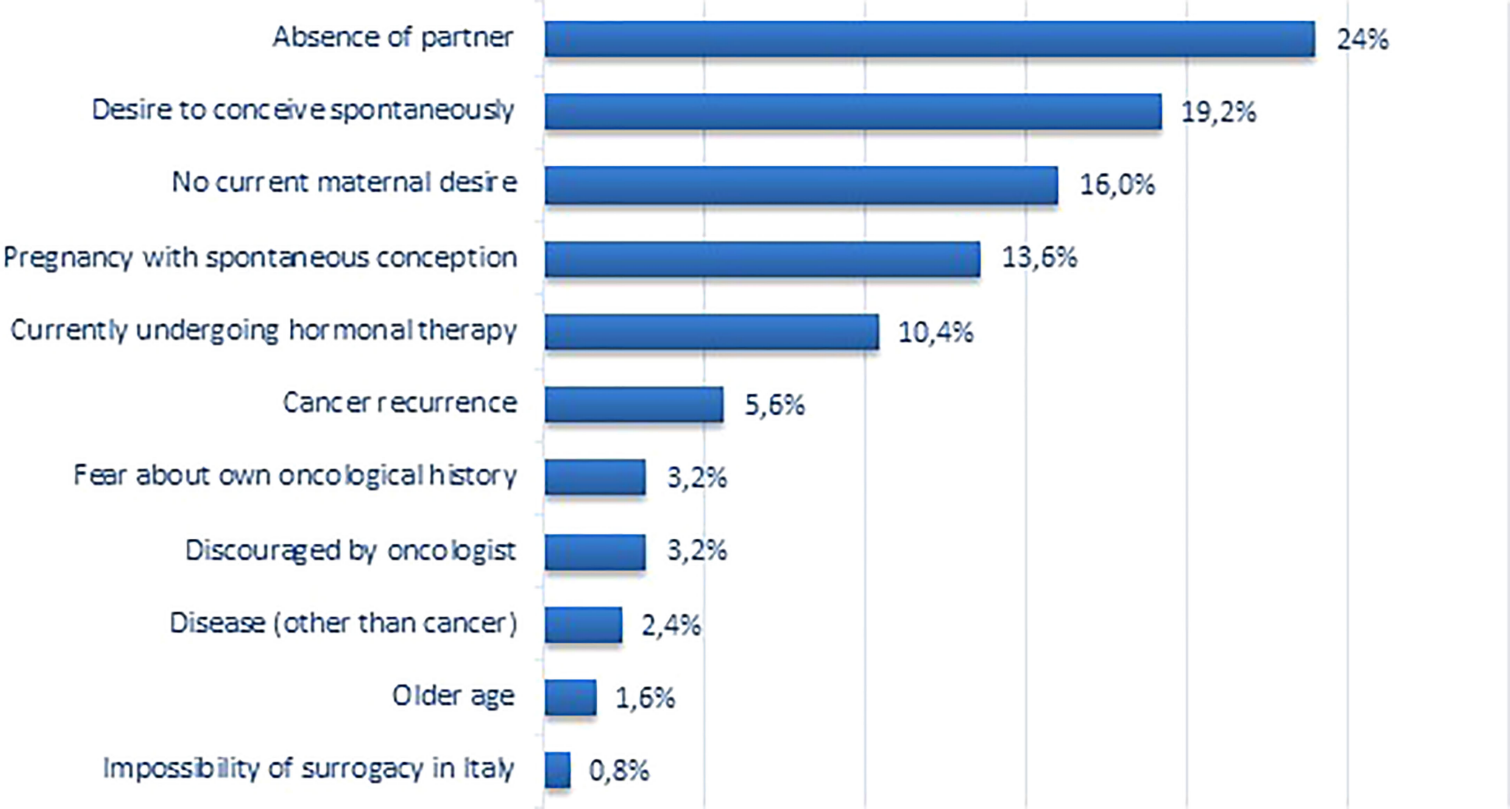
Main personal reasons reported by patients who did not return to use their frozen oocytes.

When asked about their personal reasons for not coming back (**Figure 2**), most patients reported that the absence of a partner was the principal cause (30, 24.0%), followed by the wish for spontaneous motherhood (24, 19.2%). Indeed, 24 patients (19.2%) were attempting to achieve a spontaneous pregnancy when investigators contacted them. Sixteen patients (12.8%) answered that their primary reason was that they had a spontaneous pregnancy after FP procedures. Ultimately, 13 patients (10.4%) reported that the principal reason was the ongoing hormonal therapy for breast cancer; yet, 34 (27.2%) of non-returned patients were still undergoing this type of treatment.

It is interesting to cite the cases of two patients who explicitly reported that they could not return to attempt pregnancy at Humanitas Research Hospital’s Fertility Center because of the limitations that Law 40/2004 imposes on ART in Italy.

Three patients added a secondary reason for their non-return. A patient with recurrent disease said she also had no partner, while two patients reported concerns about their age. The primary reasons indicated by the latter were the fear of disease recurrence and the oncologist’s veto about seeking pregnancy.

When specifically questioned about their intentions regarding their stored oocytes, 46 patients (36.8%) said that they were not planning to come back to use them, while 4 patients (3.2%) were unsure about this possibility. Nonetheless, all of them expressed their will to keep the storage, though they may not use it.

## Discussion

This retrospective study reports the experience over 17 years of the Humanitas Research Hospital’s Fertility Center with oncological patients undergoing oocyte cryopreservation for FP. The main goals were to evaluate the patients’ return rate and to assess the reasons influencing those who did not return. During the observation period, 11.7% of patients returned to attempt pregnancy. This come-back rate is relatively higher than the one reported in the literature and by the ESHRE consortium (ranging between 5% and 8%) (5–8). Among the reasons that more strongly influenced patients’ return, the most significant was the absence of a partner. Almost one-fourth of patients accounted for it to be the leading cause and, indeed, being single was the most significant difference between returned and non-returned patients. Younger age at freezing was another significant difference between the two groups. In a previously published study, the Humanitas Research Hospital’s Fertility Center’s research group considered the 244 patients who underwent oocyte retrieval and storage from January 2001 to March 2019. Comparing the current study’s results with that previous experience, a significant improvement in patients’ come-back rate was observed (11.7% vs. 4.5% in two years) (9). The raise in the come-back rate in the same study population after a few years may be explained by an improved awareness in clinicians, both oncologists and gynecologists, on the importance of childbearing and its positive psychological aspects on post-oncological patients. As previously stated, the present come-back rate is relatively higher than the ones found in the literature (5–8). In a study published in 2018, Cobo and colleagues compared patients who had undergone non-medical FP with oncological-FP. They reported a return rate of 12.1% among non-medical FP patients, which is similar to the one described in this study (7). This could indicate that oncologists of the Humanitas Research Hospital’s Cancer Center mainly refer patients with good prognoses (patients’ mortality rate was 5.3%) who are highly motivated to undergo fertility preservation. The fact that, at cancer diagnosis, patients who later returned to attempt a pregnancy were significantly older than those who did not come back could indeed be a drive for a stronger motivation. It is also possible to hypothesize that such a return rate might result from a good patient selection by the Fertility Center’s physicians based on patients’ age and ovarian reserve. Indeed, only 43.1% of the patients referred for FP counseling eventually had their oocytes retrieved and stored.

A cancer diagnosis in a relatively young woman has an enormous impact on her life’s planning and prospective and might completely overturn one’s life priorities. Indeed, 16% of patients said that they did not have any maternal desire at the time of follow-up. One could speculate this to be directly caused by the overwhelming effects of a cancer diagnosis, which forces women to reconsider their life goals. Specifically, the veil of uncertainty that a malignancy lies may hinder maternal sentiments, as patients feel the necessity to be more focused on themselves rather than taking care of a hypothetical child.

Modern society’s demands force a growing number of women of childbearing age to delay motherhood (11). Cancer patients then add to this all the related problems to their condition. The age at which patients return to use their oocytes is much higher than the national research average for first pregnancy (31.5 years according to the Italian national institute of statistics registry). The percentage of single women in the non-return group is significantly higher than the national population (47.7% of the Italian women are married and only 10% of the women between 25 and 49 years live single person family) (24).

Younger age at cancer diagnosis also translates into a possible higher probability of getting pregnant by natural conception, which occurred in 13.6% of the non-returned patients. This is something that has been comprehensively studied in the literature. For example, in a study published in 2009, Green and colleagues assessed different fertility indicators in a group of patients participating in the Childhood Cancer Survivor Study (CCSS). They aimed to determine the effect of oncological treatment on ovarian function and reproductive outcomes in young cancer patients. Of the 6494 female cancer survivors who participated in their survey, 1915 reported 4029 pregnancies (25). POI’s prevalence was the same as the one reported in the current study, as premature menopause occurred in 8% of patients (25).

The fact that a higher number of patients did not have a partner at follow-up is another example of the effect that a diagnosis of malignancy has on a woman’s personal life. In a prospective follow-up study performed in a single center in Dresden, Germany, Goeckenjan and colleagues submitted a questionnaire to women who had received FP counseling 3 and 6 years after the diagnosis of cancer (10). Patients were asked about their fertility, partnership, family planning, and pregnancy history (10). Most of them ascribed their non-return to the absence of a stable partnership, the fact that they had already completed their family planning, their advanced age, their fear of cancer relapse, and their fear of having a diseased child (10). These results are similar to the ones reported in the current study.

The impact of a cancer diagnosis on a woman’s family planning and future perspective was recently highlighted in a population-based analysis conducted by Anderson and colleagues that investigated the number and the timing of pregnancy and live birth after cancer diagnosis in all women diagnosed with cancer before the age of 40 years in Scotland between 1981 and 2012 with no previous pregnancies (26). The study considered 10267 cancer survivors and, by matching them with three population controls, demonstrated a reduced chance of live births and a reduced family size in those women who achieve pregnancy after diagnosis (26).

It is relevant to underline that these results were obtained in a Fertility Center based in Italy, where Law 40/2004 prohibits cryopreservation of embryos and allows the use of gametes from donors only for infertile heterosexual couples (27). This leaves single women out, forcing them to give up their maternal desire or undergo ART procedures in another country, with the economic and psychological burden that might follow. Indeed, two patients in this study had concerns about this. One of them had been hysterectomized and specifically reported being unable to have a surrogate pregnancy in Italy.

Particular attention should also be given to breast cancer patients. Indeed, premenopausal women with hormone-receptor-positive breast cancer generally receive 5-10 years of adjuvant hormonal therapy. During this period, pregnancy is contraindicated. In this study’s cohort of non-returned patients, 80.0% of breast cancer patients had received or were concurrently receiving this type of treatment. It is reasonable to believe that such a regimen delays patients’ projects and family planning. For this reason, an Italian study initiated in 2014, known as the POSITIVE trial, is investigating the impact of temporary hormonal therapy interruption to allow pregnancy (28) with *in-itinere* promising results.

The key strength of this study relies not only on the analysis of 17 years of fertility preservation in cancer patients but also on the possibility that investigators had to explore patients’ motivations for their non-return directly. Not only was it possible to analyze the objective characteristics of these patients, but also to directly take note of their own experiences and thoughts.

A limitation of the study is the not high number of patients retained in absolute terms from a single center, however, the data currently available on oncological patients underwent FP are very limited and mostly reported in case reports and case series. Therefore, multicenter and international studies are desirable in the future in order to increase the knowledge of this patient setting. Given the limitations imposed by Law 40/2004 on ART, this setting may limit any possible adaptation of the study to other countries, where different legislations apply.

## Conclusions

Nowadays, many cancer patients can plan a family after surviving their disease, as present anticancer treatments have led to high survival rates in young oncological patients. For this reason, FP techniques at the time of diagnosis before initiating gonadotoxic treatments have become standard procedures. Nonetheless, follow-up data after counseling for fertility preservation are still anecdotal.

Family planning after cancer is a complex construct, and many factors come into play in this decision. FP techniques increase the chance for a woman to have her own child after cancer, but several other factors may outweigh the biological effects. The impact of a cancer diagnosis on a woman’s maternal desire, sentimental status and life priorities should be studied more thoroughly.

Moreover, it is important to encourage studies investigating hormonal therapy suppression in breast cancer patients seeking pregnancy to reduce their time to pregnancy.

Finally, the relatively low return rates reported in oncological patients who turned to FP at the time of their cancer diagnosis may motivate some to rule out FP as a “worthy” procedure. However, the unique opportunity given by this study in offering a direct confrontation with patients on the matter underscores the importance of such procedures. Patients reported that the reassurance of having a few of their oocytes stored and preserved helped them better coping the psychological distresses caused by their disease.

## Data availability statement

The raw data supporting the conclusions of this article will be made available by the authors, without undue reservation.

## Ethics statement

Written informed consent was obtained from the individual(s) for the publication of any potentially identifiable images or data included in this article.

## Author contributions

IV and ZF wrote the research project and analyzed data. SC, BA, CA, and CF contributed to data extraction, references, and follow up analysis. ME performed the statistical analysis. RC contributed to the manuscript preparation. L-SE prepared the final draft. All authors contributed to the article and approved the submitted version.
